# Modelling of surface river plume using set-up and input data files of Delft-3D model

**DOI:** 10.1016/j.dib.2020.105899

**Published:** 2020-06-23

**Authors:** Juan Gabriel Rueda-Bayona, José Horrillo-Caraballo, Tatiana R. Chaparro

**Affiliations:** a Universidad Militar Nueva Granada, Engineering Faculty, Civil Engineering, Water and Energy (AyE) Research Group, Carrera 11 No.101- 80, Bogotá, Colombia; b Zienkiewicz Centre for Computational Engineering, College of Engineering, Swansea University, Swansea, United Kingdom

**Keywords:** Numerical modelling, Hydrodynamic, Delft3D, Waves, Wind, Tides, Currents, Sediments

## Abstract

This data article presents set-up and input data files to model a surface river plume through curvilinear nested grid in double-way mode. The hydrodynamic modelling in river deltas with intense transport processes and complex bathymetry such as the Magdalena River delta, requires a mesh grid that ease the natural river discharge into the ocean. The aforementioned may be challenging due to the numerical scheme and stability restrictions of the numerical models that difficult having efficient and effective validated simulations. This dataset files are a reference to perform analysis of the hydrodynamic river deltas, meaningful for optimizing time and resources, easing the planning of measurement campaigns what reduce risks of the personnel and instrumentation during equipment deployment and field work .The application of the set-up and input data files of this data article is shown in Rueda-Bayona et al. [1].

Specifications table

**Table:** 

Subject	Ocean engineering
Specific subject area	Hydrodynamic and transport modelling of river delta.
Type of data	Set-up files and input files.
How data were acquired	Implementation and numerical simulation in a Windows Core i7 computer with x64 bits.
Data format	Raw
Parameters for data collection	The considered conditions for using the data files were the assesment of model performance (calibration-validation) through R^2^ correlations, p-value significance, time-series analysis and feasible model parameters reported in the literature.
Description of data collection	The set-up files were configured, implemented and run considering numerical restrictions of the Delft3D model such as numercial stability, limitations of boundary conditions and feasible values of physical parameters (constants, roughness and viscosity). The set-up files are in American Standard Code for Information Interchange (ASCII) format with several file extensions according the model requirements.
Data source location	The study area is the Magdalena River delta located in 11.106909° N and −74.850756° W.
Data accessibility	Supplementary material alongside the online version of this data article.
Related research article	Selection of JONSWAP Spectra Parameters During Water-depth and Sea-state Transitions, J. Waterw. Port, Coastal, Ocean Eng. in press (2020) [1]

## Value of the Data

• The data (set-up and input files) allow to simulate currents, waves, salinity, temperature and sediments of a tropical river plume.
• The data is useful to estimate surface hydrodynamics and transport for the most important river delta in Colombia (Magdalena River).
• The utilized files can be used as reference to perform preliminary feasibility assessment of hydraulic engineering and environmental research projects.

## 1 Data description

**Fig. 1:**
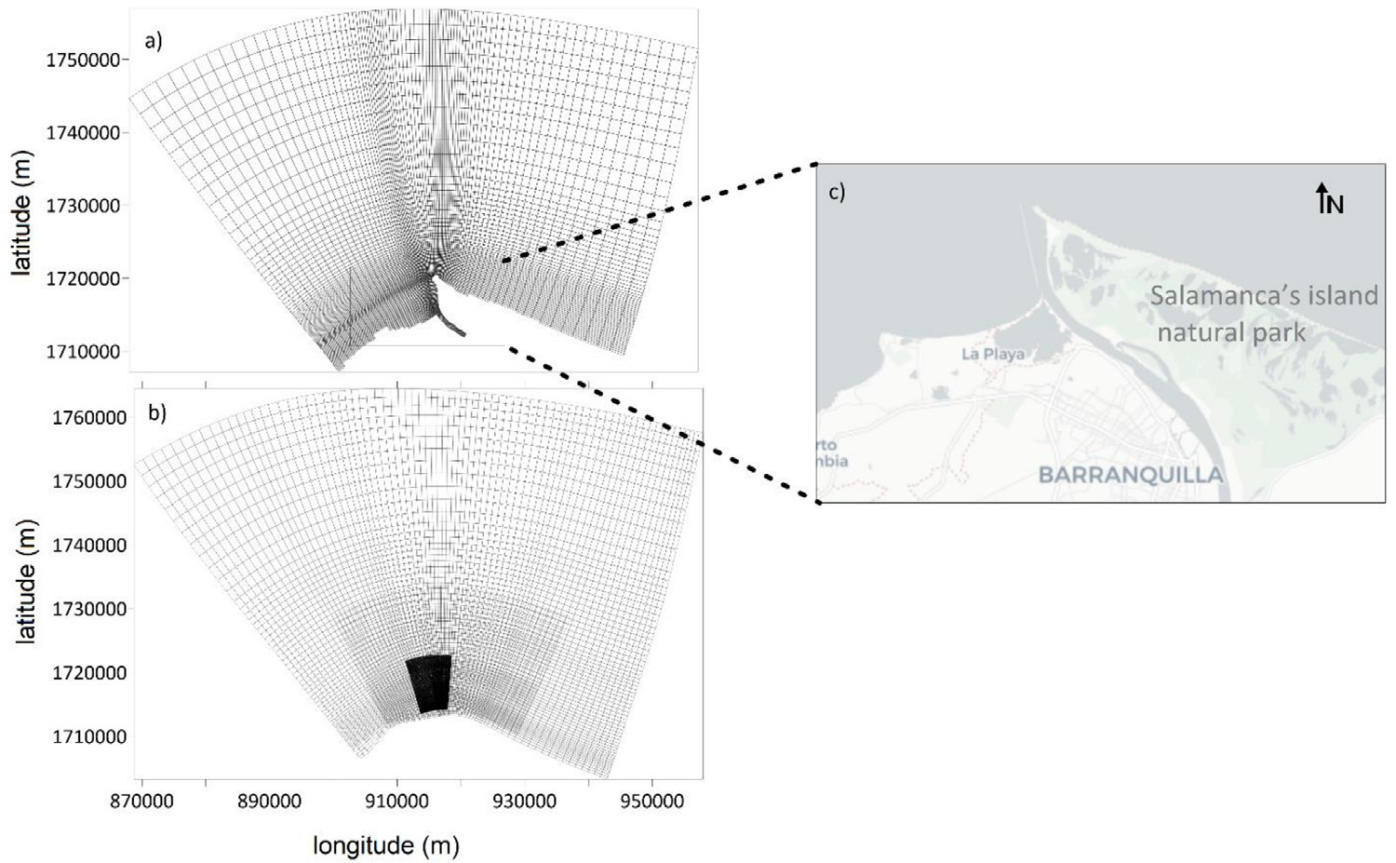
Numerical meshes: (a) hydrodynamic grid for flow module, (b) three nested grids for wave module, (c) zoom in of the Magdalena River delta. Coordinates system in Bogotá Central Magna-Sirgas.

**Fig. 2:**
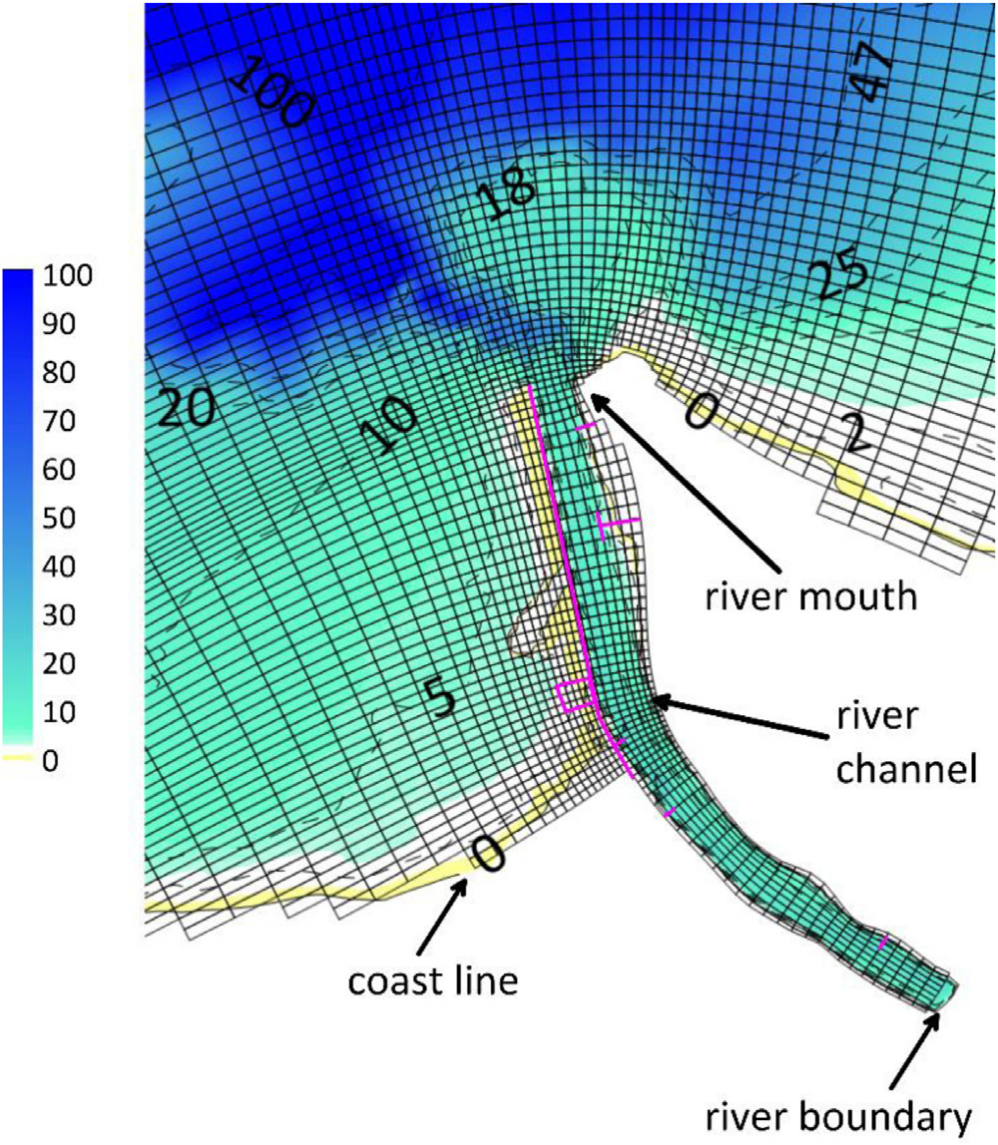
Grid cells, bathymetry and location of main features of the Magdalena River delta where label numbers indicate the isobaths. The magenta lines are model's thin dams that represent rigid structures such as dikes. Projected coordinates in Magna-Sirgas Bogota Zone (units in meters).

The setup and input data files (Table 1) are stored in a folder named as River_Delta, which gathers the information of boundary conditions, numerical and physical parameters, transport processes, observation points and domain of the study area (Figs. 1 and 2) to be simulated through the Delf3d model.

**Table 1:** Set-up and input data files of the numerical model.

Data file	Model module	File name
Master Definition Flow (MDF-file)	Flow	River_delta.mdf
Master Definition Wave (MDW-file)	Wave	River_delta.mdw
Hydrodynamic grid	Flow	River_delta.grd
Wave grid	Wave	outside_swan.grd
Bathymetry	Flow and Wave	River_Delta.dep
Boundary definition	Flow	River_Delta.bnd
Time-series flow conditions	Flow	River_Delta.bct
Transport conditions	Flow	River_Delta.bcc
Heat flux model data	Flow	River_Delta.tem
Wind data	Flow	River_Delta.wnd
Observation points	Flow and Wave	River_Delta.obs
Wave boundary condition	Wave	TPAR.bnd
Land boundary file (river and coast line)	Flow and Wave	River_Delta.ldb

## 2 Experimental design, materials, and methods

The study area is located in the Magdalena River delta (Fig. 1c) which is considered strategic for protecting the coastal-marine ecosystems of the Salamanca's island natural park and the economy development of the Barranquilla city according to Alvarado [2] and the Colombian port and maritime administration DIMAR (Maritime General Direction (www.dimar.mil.co). Then, this data article may be considered as reference to simulate complex hydrodynamic process seen in river deltas with scarce available in-situ data because of the databases’ restrictions and instrument deployment.

This data article optimized the grids and model set-up [1,^3^,4] and defined a curvilinear mesh with 3 sigma layers for the flow module, with cell size about 30 m × 30 m at the river mouth, and cells with dimensions of 550 m × 210 m for the outer area (Fig. 1a). The wave model has three nested grids: the biggest is 1.8 km × 1.8 km, the intermediate is 600 m × 600 m (Fig. 1b), and the smallest is similar to the hydrodynamic grid cells nearby to the Magdalena River mouth (Fig. 2).

The bathymetry data (Fig. 2) was extracted from the ETOPO1 database [5] and combined with data of local nautical charts. The salinity and temperature data required by the model boundaries were acquired from the World Ocean Atlas 2013 data base (www.nodc.noaa.gov). The surface data (Total Suspended Sediments, Salinity, Temperature) for the river boundary belong to the water quality monitoring data system (REDCAM, in Spanish) data base (http://siam.invemar.org.co/redcam). Climate data such as solar radiation, winds, clouds, relative humidity and air temperature for the heat flux model was obtained from the NCEP North American Regional Reanalysis (NARR) data base [6]. The information of waves and tides for the ocean boundary was retrieved from the WAVEWATCH III model (https://polar.ncep.noaa.gov/waves/) and GRENOBLE model [7] respectively. River flow data of 2010 year was provided by the IDEHA institute (https://www.uninorte.edu.co/web/ideha/sobre-nosotros). The model calibration and validation is reported in Rueda-Bayona [1] which considered numerical modelling restrictions and recommendations reported for the study area [8], [9], [10].

The main files of the dataset of this article are organized and described as follows:

In order to improve the numerical results or reducing the computational time, this data article suggest the following values for the set-up files:

1- Time step: 0.1 – 1 min.
2- Numerical parameters (Depth at grid cell faces): Mean / Mor.
3- Bottom roughness: constant or a *rgh* file with low Chezy values in boundary cells when instabilities or atypical current velocities appear.
4- Number of layers: 3 layers with 5% of thickness for the surface layer.

Because the available wave data (WAVEWATCH III) for the open boundary is located several kilometers away from the river mouth, it was necessary the utilization of a big-coarse grid for the flow and wave modules (fig 333). As a result, after each run the Delft3d (version 4.01.00) reported in the command window a finished simulation with the message “*** ERROR Deltas > water depth in TRATUR”. Then, this article verified that prompt message according the recommendations of the website's forum [11] of Delft3d model. After several inspections and tuning to the model set-up the message kept because the bathymetry in the study area strongly varies naturally, what is a cause of the prompt message, however, the numerical results were inspected and may considered good because they kept similar to the numerical results aforementioned [1], [2], [3].

## CRediT authorship contribution statement

**Juan Gabriel Rueda-Bayona:** Writing - original draft, Conceptualization, Methodology, Software, Validation, Formal analysis. **José Horrillo-Caraballo:** Writing - original draft. **Tatiana R. Chaparro:** Writing - original draft.

## Declaration of Competing Interest

The authors declare that they have no known competing financial interests or personal relationships which have, or could be perceived to have, influenced the work reported in this article.

## Appendix Supplementary materials

Image, application 1
